# Psychometric Properties of the Sensory Processing and Self-Regulation Checklist: English Version

**DOI:** 10.1155/2021/6658786

**Published:** 2021-02-20

**Authors:** Ivan Neil B. Gomez, Angelika Pauline Calsa, Jerika Toni Esguerra, Prince Joseph Heric Penetrante, Kenneth Porlucas, Maria Erica Santos, Carla Beatrice Umali, Cynthia Y. Y. Lai

**Affiliations:** ^1^Center for Health Research and Movement Science, College of Rehabilitation Sciences, University of Santo Tomas, Manila, Philippines; ^2^Department of Occupational Therapy, College of Rehabilitation Sciences, University of Santo Tomas, Manila, Philippines; ^3^Department of Rehabilitation Sciences, Hong Kong Polytechnic University, Kowloon, Hong Kong SAR, China

## Abstract

**Background:**

Sensory processing supports children's development and abilities to participate in activities across contexts. Self-regulation skills may influence how children process various sensory experiences in daily life activities. The Sensory Processing and Self-Regulation Checklist (SPSRC) is a 130-item caregiver-reported checklist, covering children's essential sensory processing and self-regulation performance in daily activities.

**Objectives:**

This study examines the psychometric properties of the SPSRC (English version) in measuring the sensory processing and self-regulation abilities of children.

**Methods:**

A preliminary field testing of the SPSRC-English was conducted in a sample of *n* = 194 children (164 without disability and 30 with a disability) to evaluate its reliability and validity properties.

**Results:**

The SPSRC-English was shown to have high internal consistency and test-retest reliability; and good discriminant, structural, and criterion validity in the sensory processing and self-regulation abilities of children with and without disability ages 4-12 years.

**Conclusion:**

The current study provides initial evidence on the reliability and validity of SPSRC-English in measuring the sensory processing and self-regulation abilities in children with and without a disability. The SPSRC-English may provide salient information supporting the understanding of sensory processing difficulties among children.

## 1. Introduction

Sensory processing involves the central and peripheral nervous systems in processing external stimuli from their senses [1–4]. It is the ability to register and manipulate information and to make sense of different types of received sensations [5, 6]. The processing of these sensations serves as the underlying basis for further acquisition of more complex skills that support participation in daily functioning [7, 8]. Some children may encounter difficulty in processing sensory stimuli, which may impair their roles and participation in daily living. Sensory processing problems occur in both children with and without disabilities [9]. The incidence of sensory processing difficulties has been suggested to be around 40-90% among children with disabilities and as much as 5-23% in typically developing children [5, 10–14]. Diagnosis of a sensory processing disorder involves the presence of several sensory behaviour symptoms and a significant impact on the child's functioning and participation [15]. Despite its prevalence and influence, the diagnosis of sensory processing disorders remains inconclusive [4, 16, 17].

Over the past years, several attempts have been made to classify sensory processing disorders [2, 4, 6, 9, 18]. Miller et al. [16] proposed a nosology for classifying sensory processing disorders. Sensory modulation disorder is one of the patterns and is described as the difficulty in regulating different sensory inputs (i.e., tactile, vestibular, proprioceptive, etc.) from daily activities. Sensory modulation disorders may present as sensory overresponsiveness, underresponsiveness, or seeking/craving. These unusual responses may be from an underlying deficit in the regulation of one's neurophysiological systems [2, 19, 20]. Differences in the neurophysiological and behavioural response to sensory stimuli may also be linked to cultural and genetic factors [21, 22].

Self-regulation is the ability to change one's self according to the internal and external environments [23]. To interact with the environment successfully, an individual needs to be able to change and respond accordingly [24]. Fundamental capacities are needed for self-regulation. For self-regulation to develop, the underlying neurophysiological modulation must be well developed to support behavioural regulation [25, 26]. Self-regulation difficulties and sensory processing disorders may typically coexist [27]. Children with disabilities have been reported to exhibit to manifest an overlap of these behavioural symptoms [27, 28]. While sensory processing and self-regulation are interlinked [27, 29], it is still essential to examine any possible factor that contributes to the behavioural pattern in the sensory events for each individual [30]. A measure that incorporates aspects of self-regulation to clarify how children regulate sensory responses is needed.

The Sensory Processing and Self-Regulation Checklist (SPSRC; [30, 31]) is a parent/caregiver-answered questionnaire that was developed to examine sensory processing and self-regulation in children (typically developing and with disabilities) aged 3–8 years. The SPSRC is a single instrument for the evaluation of sensory processing and self-regulation difficulties in children. The SPSRC comprises two sections: self-regulation ability and sensory processing ability. The self-regulation ability section is aimed at identifying children's difficulties with behavioural regulation and comprises items that reflect the ability to self-regulate. The sensory processing ability section is aimed at identifying behavioural patterns in response to sensory input and at quantifying the extent of difficulties encountered by children upon receiving different types of sensory input. The SPSRC has been proven to have good reliability and validity in examining sensory processing and self-regulation in children [30]. The original SPSRC items are written in Chinese. However, through a multiprocess of language equivalency, an English version is currently available [32]. While useful for bilingual countries, it cannot be assumed that its original psychometric properties remain intact. The psychometric properties of a translated questionnaire should be deemed relevant to the population and context to whom it is intended to be used [33].

This research is aimed at testing the reliability and validity of the SPSRC-English version. The information obtained from the SPSRC-English may supplement the current understanding of sensory processing difficulties among children.

## 2. Materials and Methods

### 2.1. Participants

We recruited *n* = 194 (164 without disabilities and 30 with disabilities) Filipino boys and girls to participate in this study. For children without disabilities, they must be enrolled in a regular classroom, appropriate to their chronological age. A history of a parent-reported medical condition (i.e., physical, developmental, intellectual, behavioural, etc.) or failed grade level warranted exclusion. Children with disabilities were identified based on the submitted medical, educational, or legal report. The person most knowledgeable (i.e., spends the most time with the child) was asked to complete the questionnaire. The participants are of Filipino descent, have completed at least secondary school, and bilingual (i.e., Filipino and English). Ethical approval was obtained from the Ethics Review Committee of the College of Rehabilitation Sciences, University of Santo Tomas.

### 2.2. Instruments

#### 2.2.1. Sensory Processing and Self-Regulation Checklist (SPSRC)

The SPSRC [31] was developed as a single checklist that provides information on both sensory processing and self-regulation for children 3 to 8 years of age. The 130-item checklist has two parts subdivided into several sections and factor scales. Part 1 (37 items) looks into self-regulation and has three sections ((1) physiological, (2) social/cognitive/emotional, and (3) facing changes or challenges) and four factor scales ((1) emotional regulation, facing challenges; (2) emotional regulation, facing changes; (3) physiological regularity and response to soothing; and (4) autonomic activity). Part 2 (93 items) consists of items related to sensory processing, further divided into six sections ((1) auditory, (2) visual, (3) tactile, (4) gustatory/olfactory, (5) vestibular, and (6) proprioceptive) and four factors ((1) sensory-seeking behaviour, (2) sensory underresponsivity, (3) sensory overresponsivity, and (4) stability of sensory responsivity). The parents are instructed to report on their child's typical performance within the last three months on the items on the checklist using a 5-point Likert scale (5: never, 4: seldom, 3: sometimes, 4: most of the time, and 1: always) with some items having reversed scoring. A higher score denotes a more favourable performance. The original version is in Chinese with a currently available English-translated version. Acceptable psychometric properties have been reported for the SPSRC [30, 31]. The English version [32] was translated from the original HK-Chinese version through a rigorous process of forward and backward translation and an expert panel that reviewed the equivalency of the translated items.

#### 2.2.2. Short Sensory Profile (SSP)

The SSP was developed as a parent/caregiver-reported measure of children's ability to process sensory information in daily life activities for ages 3-10 years old [34] and was based on the original Sensory Profile [35]. Both iterations use normative data to ground its classification system. The parents/caregiver rate the child on each item using a 5-point Likert scale where a lower score indicates less favourable performance. The SSP is a reliable and valid tool to look at children's sensory processing issues that affect their performance [35].

#### 2.2.3. Sense and Self-Regulation Checklist (SSC)

The SSC was developed to be a parent/caregiver measure of comorbid symptoms related to sensory processing and self-regulation in regular daily life situations for children with autism under the age of six [29]. SSC comprises six sensory subdomains (touch-pain, auditory, visual, taste-smell, hyperreactive to noninjurious stimuli, and hyporeactive to injurious stimuli). Parents/caregivers are instructed to answer each item using a 3-point Likert scale with a higher score reflecting less favourable performance. The SSC has been suggested to have reliable and valid psychometric properties [29] and was used in this study for convergent validity testing of the SPSRC-English.

### 2.3. Procedures

We recruited participants through verbal and/or written communication with various schools. These institutions and individuals received the study information packet, which contains an abridged version of our research proposal, invitation letters to the parents, and the relevant forms and questionnaires. Parents who consented to participate received individual research packets containing the relevant materials and were asked to rate their child's performance in the last three months on questionnaire items. After 1-2 weeks, the informed consent form, demographic questionnaire, and the SPSRC-English were collected from the parents who participated in this study. A conveniently sampled subgroup of the parents was sent the SPSRC-English after two weeks and given similar earlier instructions.

### 2.4. Data Analysis

Data from the participants were initially encoded in MS Excel and later on anonymized in the SPSS ver. 23.0 software, which also served as the statistical software used to determine the psychometric properties of the SPSRC-English. We evaluated the psychometric properties of the SPSRC-English by examining its reliability and validity.

We tested reliability properties by evaluating the internal consistency and test-retest reliability within the section scores (Parts 1 and 2) and composite score (Total) of the SPSRC-English using Cronbach's *α*. A minimum threshold of >0.70 was placed to indicate an acceptable internal consistency [36].

Validity properties were tested by specifically looking into the construct and criterion validity of SPSRC-English. Construct validity testing indexed the discriminant, structural, known-group validity. Discriminant validity is examined by using Pearson's correlation coefficient. Structural validity was evaluated by evaluating the subscales and factor scales within each part using intraclass correlation statistics. Known-group validity was tested by comparing group differences between disability status, gender, and age differences on the part, subscale, factor, and composite score on the SPSRC-English using an independent *t*-test and analysis of variance, with *α* = 0.05. Criterion validity is examined with concurrent validity testing by comparing the relevant part, subscale, factor, and composite scores of SPSRC-English with the SSP and SSC.

## 3. Results

### 3.1. Participant Demographics

We recruited *n* = 194 (164 without disabilities and 30 with disabilities) school-aged children with a mean age of 7.88 (1.83) years in this study, with an age range of 4-12 yrs. There were slightly more males (52.10%) in this sample compared to females (47.90%). The specific diagnosis of the sampled children with disabilities varied.  Table 1 presents a summary of the demographics of the participants in this study.

### 3.2. Internal Consistency

The consistency of responses to the items of the SPSRC-English was tested to determine whether each of the subscale and composite scores measured the same general construct. Cronbach's *α* coefficients were 0.94 and 0.99 for the items in Part 1 (37 checklist items) and Part 2 (93 checklist items) of the SPSRC, respectively. Overall Cronbach's *α* coefficient for the SPSRC composite (130 checklist items) was 0.99.

### 3.3. Test-Retest Reliability

The test-retest validity of SPSRC-English was evaluated by comparing Parts 1 and 2 and Total scores on two separate occasions among a conveniently sampled *n* = 20 children without disability. The parents or caregiver of the sampled children answered the SPSRC-English twice with a two-week interval for each measurement (the same parent/caregiver answering the checklist). The test-retest coefficient represented by Cronbach's *α* was 0.93 for Part 1, 0.96 for Part 2, and 0.92 for the Total score.

### 3.4. Discriminant Validity

The discriminant validity was examined by testing the relationship between the mean scores of Part 1 (self-regulation ability) and Part 2 (sensory processing ability) of the SPSRC-English. The Pearson correlation coefficient was *r* = 0.70 (*p* = 0.002).

### 3.5. Structural Validity

The structural validity of SPSRC-English was examined using intraclass correlation statistics for the subscale (*r* = 0.83, *p* < 0.001) and factor scale (*r* = 0.53, *p* < 0.001) of Part 1 and was found to be significant. Similar significant results were also obtained for the subscale (*r* = 0.88, *p* < 0.001) and factor scale (*r* = 0.35, *p* < 0.001) of Part 2.

### 3.6. Known-Group Validity

Data from *n* = 164 children without disability from different age groups (4-12 yrs.) were compared on their SPSRC-English mean scores (part, subscale, factor, and composite) using a one-way ANOVA test. The results reveal significantly different SPSRC-English scores across age groups, specifically for Part 1: self-regulation abilities (*p* < 0.001, *d* = 1.92), Part 2: sensory processing abilities (*p* < 0.001, *d* = 2.58), and overall ability (*p* < 0.001, *d* = 2.82).

Gender differences (*n* = 164 children without disability: 82 females and 82 males) in the SPSRC mean scores (part, subscale, factor, and composite) were likewise compared, and the results show that overall, males have higher scores compared to females. However, the *t*-test results reveal that these differences are not significant, except for subscale 3: facing challenges (*p* = 0.03, *d* = 1.03) and factor 4: autonomic activity (*p* = 0.04, *d* = 1.05) of Part 1 (self-regulation ability).

We compared the SPSRC-English mean scores (part, subscale, factor, and composite) between an age and gender-matched sample of 30 children without disability and 30 children with disability using an independent *t*-test (Table 2). The results indicate significant differences in self-regulation ability (*p* < 0.001), sensory processing ability (*p* < 0.001), and overall ability (*p* < 0.001), with children without disability showing higher scores indicative of favourable performance.

### 3.7. Concurrent Validity

Mean scores (part, subscale, factor, and composite) of *n* = 30 children with disabilities ages 4-12 years on their SPSRC-English were examined on their concurrent relationship to respective scores on the SSP with SSP and SSC (Table 3). The Pearson correlation between the total scores on both the second part of SPSRC-English and the SSP is significantly strong (*r* = 0.77, *p* = 0.002). The significant correlation between the second part of SPSRC-English and SSP sensory domains ranged from 0.63 to 0.77. For factor score correlations, only the SPSRC-English factor scores for sensory-seeking behaviour and SSP factor score for underresponsive/seeks sensation reached significant moderate correlation (*r* = 0.68, *p* = 0.04).

The Pearson correlation between the total scores of the second parts of SPSRC-English and SSC (sensory domain) is significantly strong (*r* = −0.74, *p* = 0.002). The related sensory domains of SSC were significantly correlated with scores on the second part of SPSRC-English, and the results indicate moderate correlation for the domains on vision (*r* = −0.55, *p* = 0.03) and taste/smell (*r* = −0.58, *p* = 0.04). We compared relevant SSC domains with Part 1 scores of the SPSRC-English, and the results show that for the subscales, the significant correlation was between *r* = −0.62 and -0.70, while for the factor scale, the correlation ranged from *r* = −0.20 to -0.72.

## 4. Discussion

This study was aimed at examining the psychometric properties of the SPSRC-English. The various evaluation methods used demonstrate that SPSRC-English has overall satisfactory psychometric properties in measuring the sensory processing and self-regulation of children with and without disability, ages 4-12 years old. This research extends the earlier findings of the original version of SPSRC [30, 31] that found that the original version of SPSRC (HK-Chinese) has good reliability and validity to assess the same constructs, but among 3-8-year-old children with and without ASD.

Taken as a whole, the parts (Parts 1 and 2) and composite (overall ability) score of SPSRC-English have excellent internal consistency, similar with the original version in Chinese [30, 31]. The items in each part of the SPSRC-English reliably measure the fundamental capacities of behavioural regulation and behavioral responses to sensory stimuli a child encounters in daily life activities.

In assessing the stability of the responses on the SPSRC-English, the results of this study indicated excellent test-retest reliability, with time variance at a two-week interval. This finding is similar to the original version of the questionnaire [30, 31], which connotes that the SPSRC-English demonstrates the ability for parents to sufficiently measure their children's sensory processing and self-regulation behaviours over time when change is not expected.

In this research, discriminant validity was operationally defined as a measure of test validity that looks into how measurements that are not related do not correlate with each other [37]. Voorhees et al. [38] suggested a threshold of 0.85 correlation be set to determine whether two constructs overlap. Parts 1 and 2 in the SPSRC-English had a moderate correlation, below the threshold. The findings in this research provide evidence that each part of the SPSRC can discriminate scales measuring self-regulation and sensory processing abilities separately. It should be noted though that in the original SPSRC version, a different operational definition was used in defining discriminant validity.

Structural validity measures refer to whether scores within an instrument adequately parallel the underlying construct dimensions [37]. The examination of the structural validity of the subscale and factor scales for each part of SPSRC-English demonstrates the relevance of the item scores as a latent construct reflecting similar but independent dimensions. While the SPSRC-English has demonstrated that each of its parts discriminates underlying constructs, the items within each part and their consequent subscales and factor scales further reflect the valid measurement of analogous dimensions. The original SPSRC reports similar findings using a factor analysis method [30, 31]. However, this research approached structural validity using ICC to depict the strength of the relationship between the subscales and factor scales for both parts of the SPSRC-English.

Several factors may influence a child's behavioural regulation and response to sensory stimuli in daily activities. We tested three salient factors, age, gender, and disability status. There are significant differences in the self-regulation and sensory processing abilities among children from different age groups. This finding was similar to previous reports that developmental stages may influence children's self-regulation [39] and sensory processing [11, 28, 40]. While this finding warrants further studies, developmental trajectories may provide context clues in evaluating the self-regulation and sensory processing skills of children ages 4-12 years old.

Gender differences were found to be insignificant in the self-regulation, sensory processing, and overall abilities of the sampled participants in this study. This finding corroborates earlier results that boys and girls have similar tendencies in self-regulation abilities [41] and sensory processing [8, 40]. Nevertheless, subscale C (facing changes or challenges) and factor scale 4 (autonomic activity) of Part 1 of the SPSRC-English resulted in significant gender differences. Biologically related factors influencing the regulation of behavioural responses to changes and challenges may exist between boys and girls [42]. Furthermore, underlying autonomic functions has been found to significantly differ between genders [5, 43–45]. Without these dimensions in the SPSRC-English, it would have been impossible to reflect these specific gender differences.

In this research, children with disabilities were found to have significantly lower scores compared to typically developing ones on their self-regulation, sensory processing, and overall abilities. It has been reported that 40-90% of children with disabilities may display sensory processing issues [5, 10–14]. The SPSRC encompasses the fundamental capacity for self-regulation including physiological functions [30]. A child's physiological regulation of response may influence their sensory processing abilities [46], which may explain the self-regulation differences between children with and without disabilities. The varied disability conditions in the clinical group recruited in this study's sample may reflect their patterns of sensory processing difficulties [12, 14, 20, 46], which warrant future inquiry. Nevertheless, the extent of our examination has proven that SPSRC-English is sensitive to differences and similarities among the various groups tested in this research.

The various scores on the SPSRC-English were correlated to the different domains and scales of the SSP and SCC, and the results point to several significant correlations, demonstrating SPSRC-English's criterion validity. Criterion validity refers to how well scores in the instrument being evaluated adequately reflect those of a proven measure [37]. In the original SPSRC, the authors reported similar findings using its convergent validity to the Chinese Sensory Profile [30]. In this research, we explored the criterion validity using the SSP [34], the shortened version of the Sensory Profile [35] from which the SSP was based on. The correlation with the SSP indicates the SPSRC-English's adequacy to measure similar constructs.

The SPSRC is unique in its ability to singularly examine sensory processing and self-regulation among children [30]. Comparing the scores on SPSRC-English and the SSC, the results were similar to the foregoing. The correlated scores (negative correlations due to the reversed scoring of SSC compared to SPSRC) may indicate SPSRC-English's ability to measure related constructs. The SSC [29] is somewhat similar to the SPSRC in measuring sensory and self-regulation behaviours among children. SPSRC-English was shown to concurrently perform in measuring similar outcomes with a previously published one.

Thus far, the SPSRC-English has shown to have remarkable psychometric properties in measuring the sensory processing and self-regulation of children comparable to its original version. However, one apparent notable distinction should be made related to the construct development of the tool. The SPSRC-English version was developed through a multiprocess translational approach to retain its cultural constructs. The participants in this study comprised a bilingual (i.e., Filipino and English) Filipino sample, a culture that is different from the population for which the original version was intended for. On the one hand, the results of this study may connote the stability of parent-observed responses related to sensory processing [47, 48] and self-regulation [49] among children. On the other hand, several authors have implicated the importance of culture in sensory processing measures [50]. The physical objects from which sensory stimuli emanate from and its familiarity and value to an individual may vary from one culture to another [30, 51]. Even among similar cultures living in distinct countries, variations in parental observations on children's behaviours have been observed (Yung et al., 2019), which raises the issue of whether geographic environment and/or genetic predispositions may influence the regulation of responses to sensory stimuli [21, 22]. SPSRC-English's nature of data collection is dependent on the veracity of response from the children's parents and caregivers, which may be prone to subjectivity and bias. Thus, the authors of this research agree with Lai et al. [30] in recommending the use of the checklist along with laboratory-based and neurophysiological testing to provide a more precise measure of a child's skills and performance.

## 5. Conclusion

In conclusion, this study has provided evidence supporting the favourable psychometric properties of the SPSRC-English. We extend previous research on SPSRC and provide compelling proof on its high internal consistency; excellent test-retest reliability; and good structural, construct, and criterion validity in the sensory processing and self-regulation abilities in children with and without disability ages 4-12 years. The information obtained from the SPSRC-English may further supplement the current understanding of sensory processing difficulties among children.

## Figures and Tables

**Table 1:** Summary of age and gender of the participants (*n* = 194).

Demographic		*n* (%)
Gender	Female	93 (47.90%)
	Male	101 (52.10%)

Age (yrs.)	4	9 (4.60%)
	5	13 (6.70%)
	6	8 (4.10%)
	7	30 (15.50%)
	8	55 (28.40%)
	9	40 (20.60%)
	10	16 (8.20%)
	11	15 (7.70%)
	12	8 (4.10%)

Presence of disability	Without disability	164 (84.50%)
	With disability	30 (15.50%)

Types of disability	Attention-deficit, hyperactivity disorder	2 (6.67%)
	Autism spectrum disorder	6 (20.00%)
	Down syndrome	2 (6.67%)
	Intellectual disability	8 (26.67%)
	Learning disability	3 (10.00%)
	Others	9 (30.00%)

**Table 2:** Comparison of mean scores on the SPSRC-English between children with and without disability (*n* = 60).

SPSRC-English scores	Disability status		*p*
	Without *n* = 30	With *n* = 30	
*Part 1: self-regulation ability*	151.2 (20.70)	116.73 (11.74)	<0.001
Subscale			
(a) Physiological	45.97 (7.56)	35.63 (3.73)	<0.001
(b) Social/cognitive/emotional	54.80 (7.14)	41.10 (6.40)	<0.001
(c) Facing changes or challenges	50.43 (8.08)	40.00 (76.69)	<0.001
Factor scale			
(1) Emotional regulation, facing challenges	38.00 (5.75)	29.03 (3.85)	<0.001
(2) Emotional regulation, facing changes	23.60 (5.06)	19.53 (2.45)	<0.001
(3) Physiological regularity and response to soothing	48.33 (6.13)	33.10 (6.41)	<0.001
(4) Autonomic activity	41.27 (7.33)	35.07 (6.17)	0.001
*Part 2: sensory processing ability*	411.10 (43.60)	297.73 (44.13)	<0.001
Subscale			
(a) Auditory	65.87 (10.70)	41.67 (9.85)	<0.001
(b) Visual	57.80 (9.25)	37.67 (4.60)	<0.001
(c) Tactile	83.60 (11.93)	55.33 (10.24)	<0.001
(d) Taste and smell	59.60 (5.24)	47.77 (11.34)	<0.001
(e) Vestibular	78.50 (7.60)	62.20 (12.71)	<0.001
(f) Proprioceptive	65.73 (6.45)	53.10 (10.21)	<0.001
Factor scale			
(1) Sensory-seeking behaviour	151.37 (15.40)	114.20 (17.48)	<0.001
(2) Sensory underresponsivity	104.93 (12.93)	74.07 (10.87)	<0.001
(3) Sensory overresponsivity	129.13 (91.63)	91.63 (17.37)	<0.001
(4) Stability of sensory responsivity	25.67 (3.88)	17.83 (5.05)	<0.001
*Overall ability*	562.30 (62.76)	414.47 (50.50)	<0.001

**Table 3:** Correlation between SPSRC-English, SSP, and SSC scores among children with disabilities (*n* = 30).

SSP			SPSRC	SSC		
*p*	*r*	Items		Item	*r*	*p*
*Part 1*						
Subscale						
			Physiological	Self-regulation: orientation/attention/self-soothing/sleep	-0.26	0.37
				Self-regulation: toilet training	-048	0.13
				Self-regulation: digestion	-0.45	0.11
			Social/cognitive/emotional	Self-regulation: orientation/attention/self-soothing/sleep	-0.45	0.11
				Self-regulation: behaviour—irritability, aggression, self-injurious	-0.70	0.01
			Facing changes or challenges	Self-regulation: orientation/attention/self-soothing/sleep	-0.62	0.02
Factor						
			Emotional regulation, facing challenges	Self-regulation: orientation/attention/self-soothing/sleep	-0.30	0.31
				Self-regulation: behaviour—irritability, aggression, self-injurious	-0.20	0.50
			Emotional regulation, facing changes	Self-regulation: orientation/attention/self-soothing/sleep	-0.04	0.90
				Self-regulation: behaviour—irritability, aggression, self-injurious	-0.32	0.30
			Physiological regularity and response to soothing	Self-regulation: orientation/attention/self-soothing/sleep	-0.70	0.01
			Autonomic activity	Self-regulation: orientation/attention/self-soothing/sleep	-0.72	0.004
				Self-regulation: toilet training	-0.27	0.35
				Self-regulation: digestion	-0.29	0.31

*Part 2*						
Subscale						
0.001	0.76	Auditory filtering	Auditory	Hearing	-0.38	0.18
0.01	0.63	Visual/auditory sensitivity	Visual	Vision	-0.58	0.03
0.002	0.71	Tactile sensitivity	Tactile	Touch/pain	-0.41	0.15
0.07	0.47	Taste/smell sensitivity	Taste and smell	Taste/smell	-0.55	0.04
0.26	0.30	Movement sensitivity	Vestibular			
0.41	0.22	Low energy/weak	Proprioceptive			
0.002	0.77	Total	Total	Total	-0.74	0.002
Factor						
0.04	0.68	Underresponsive/seeks sensation	Sensory-seeking behaviour			
0.12	0.41	Underresponsive/seeks sensation	Sensory underresponsivity			
0.05	0.67	Visual/auditory sensitivity	Sensory overresponsivity			
0.01	0.66	Tactile sensitivity				
0.04	0.51	Taste/smell sensitivity				
0.07	0.47	Movement sensitivity				

## Data Availability

Data available upon request from the corresponding author.
